# Assistance from a mixing zone model to perform aortic femoral perfusion strategy with severe atherosclerotic and artheromic aortic disease for endoscopic minimally invasive redo mitral valve repair

**DOI:** 10.1051/ject/2024036

**Published:** 2025-03-07

**Authors:** Tomohisa Takeichi, Yoshihisa Morimoto, Akitoshi Yamada, Takanori Tanaka, Kunihiro Fujiwara, Masanobu Sato, Ryo Toma, Kiyoto Mitsui, Takumi Sugita, Hiroki Yamada, Kanako Nakagaki, Hiroto Kuriyama, Kunio Gan

**Affiliations:** 1 Department of Clinical Engineering, Kitaharima Medical Center 926-250, Ichiba-cho, Ono-shi Hyogo 675-1392 Hyogo Japan; 2 Cardiovascular Surgery, Kitaharima Medical Center 926-250, Ichiba-cho, Ono-shi Hyogo 675-1392 Hyogo Japan

**Keywords:** Cardiopulmonary bypass (CPB), Atherosclerotic and artheromic, Mixing zone, Minimally invasive cardiac surgery

## Abstract

Minimally invasive cardiac surgery (MICS) for redo mitral valve surgery in the presence of severe atheroma and atherosclerotic diseased atherosclerotic and artheromic aorta presents significant challenges and increases the risk of postoperative cerebral infarction. At our institution, to mitigate the risk of postoperative cerebral complications, we employ a strategy combining antegrade and retrograde perfusion during MICS for patients with atherosclerotic and artheromic aorta. However, the mixing zone during cardiopulmonary bypass (CPB) with combined antegrade and retrograde perfusion has not been thoroughly evaluated. In this case, we performed a completely endoscopic MICS redo mitral valve plasty (MVP). CPB was established using cannulation of both the ascending aorta (Asc Ao) and the femoral artery (FA). The patient received planned systemic hyperkalemia without an aortic cross clamp. In addition, due to aortic insufficiency, circulatory arrest was also needed. The patient experienced an uneventful post-operative recovery without any cerebral complication. Furthermore, we evaluated the mixing zone during the combined antegrade and retrograde perfusion using an arteriovenous circulation model. Our findings suggest that when performing perfusion via the Asc Ao and FA, it is advisable to select Asc Ao cannulation size reduced by one size against FA cannulation size to optimize the procedure.

## Introduction

In a recent study, minimally invasive cardiac surgery (MICS) offers distinct advantages in redo cases when compared to median sternotomy [1]. Cardiopulmonary bypass (CPB) is typically established via femoral artery (FA) cannulation in the minithoracotomy approach [2, 3]. However, retrograde perfusion is associated with a higher incidence of postoperative neurological complications compared to antegrade perfusion [4–6]. To mitigate the risk of postoperative cerebral complications, particularly in patients with a severe atherosclerotic and atheromic diseased, CPB is established through axillary or ascending aorta (Asc Ao) cannulation according to the facility’s protocol [7].

At our institution, in-patients presenting with a severe atherosclerotic and atheromic diseased, we utilize a combination of Asc Ao or axillary artery and FA perfusion. The criteria for this approach include any of the following findings on a preoperative enhanced computed tomography (CT) scan of the aorta or iliac arteries: thrombosis with a thickness greater than 4.0 mm or circumferential calcification. Based on these criteria, we employed a combination of Asc Ao and FA perfusion. Additionally, we evaluated the mixing zone when antegrade and retrograde perfusion were combined using an arteriovenous circulation system [8].

This study was approved by the Institutional Review Board of Kitaharima Medical Center (IRB 06-16), with a waiver of informed consent.

## Case report

**Figure 1:**
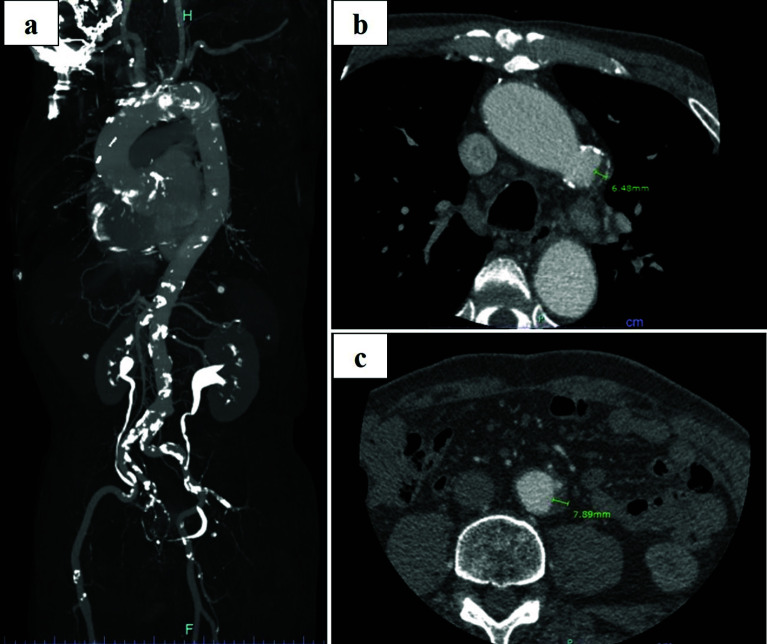
(a) Preoperative contrast-enhanced CT of the atherosclerotic and artheromic aorta. (b, c) The aortic arch and descending aorta indicate intimal 6.48 mm and 7.89 mm of intimal thickening of the aortic arch and descending aorta combined with calcification. CT: Computed Tomography.

The patient (height 164 cm; weight 54 kg) had a history of coronary artery bypass grafting (CABG) left internal thoracic artery to left anterior descending artery (LITA-LAD), aorta to radial to posterior descending artery (Ao-RA-#14), and gastroepiploic artery to posterior descending branch to atrioventricular branch (GEA-#4PD-#4AV) 4 years ago. At this time, the patient was diagnosed with severe mitral regurgitation and patent foramen ovale (PFO) by transesophageal echocardiography (TEE). The contrast-enhanced CT scan revealed calcification observed from the Asc Ao extending the abdominal aorta. Also, the aortic arch and descending aorta were observed intimal thickening >4.0 mm (6.48 mm and 7.89 mm of intimal thickening in the aortic arch and descending aorta) combined with calcification (Figures 1a–1c). The patient had a low left ventricular ejection fraction (44%) and EuroSCORE (European System for Cardiac Operative Risk Evaluation) was 13%. The totally endoscopic procedure was planned for redo-MVP under systemic hyperkalemia without cross-clamping due to being difficult aortic cross-clamping. Moreover, to prevent postoperative cerebral infarction, the Asc Ao and FA were planned to establish CPB. Table 1 indicates CPB management and postoperative outcomes including preoperative CPB plan.

**Figure 2:**
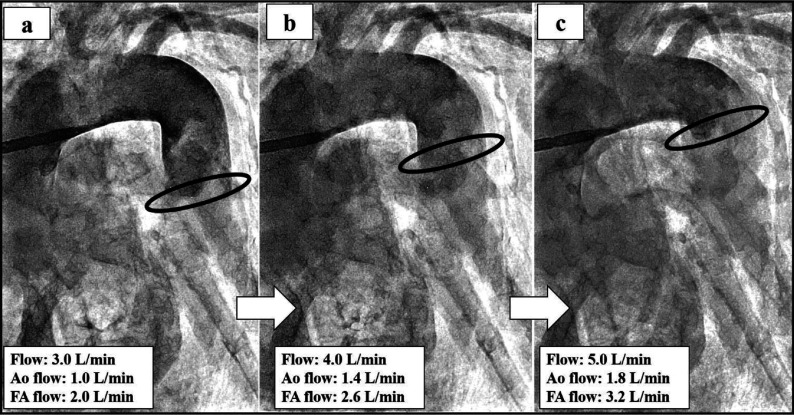
(a–c) The evaluation of the mixing zone if changing the flow rate from 3.0 L/min to 5.0 L/min by using 14Fr in the ascending aorta and 18Fr in the femoral artery. The black line indicates the mixing zone.

**Figure 3:**
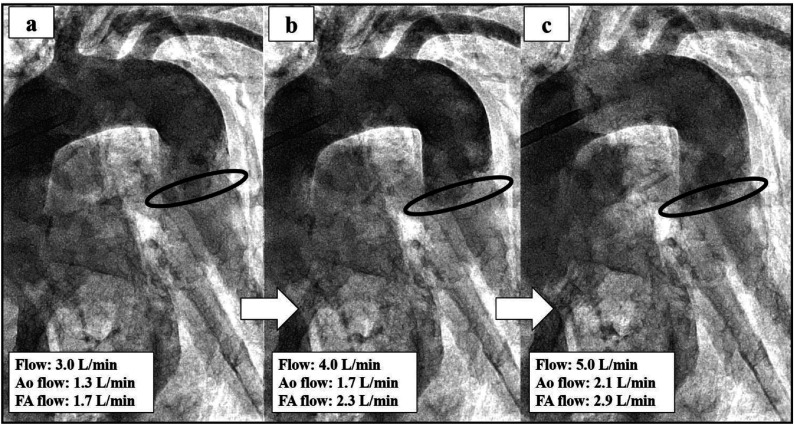
(a–c) The evaluation of the mixing zone if changing the flow rate from 3.0 L/min to 5.0 L/min by using 14Fr in the ascending aorta and 16Fr in the femoral artery. The black line indicates the mixing zone.

**Figure 4:**
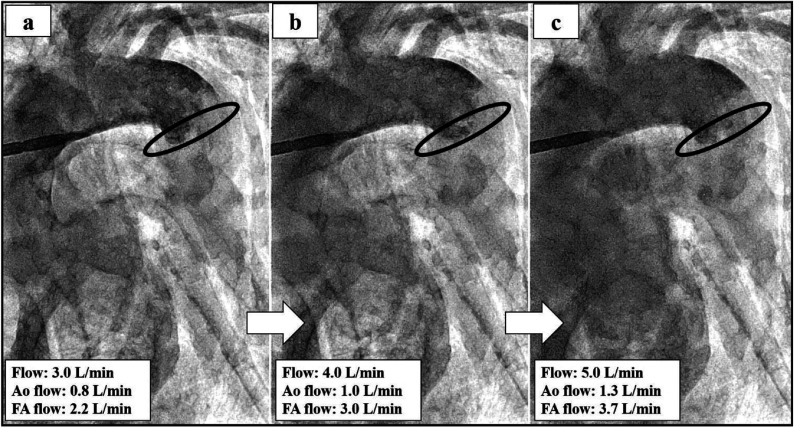
(a–c) The evaluation of the mixing zone if changing the flow rate from 3.0 L/min to 5.0 L/min by using 14Fr in the ascending aorta and 20Fr in the femoral artery. The black line indicates the mixing zone.

**Table 1:** CPB management and outcomes including preoperative CPB plan. CPB.: Cardiopulmonary bypass; Rt FA.: Right femoral artery; Rt FV.: Right femoral vein; CI.: Cardiac index; mABP.: Mean arterial blood pressure; ICU.: Intensive care unit.

CPB management and outcomes	
Preoperative CPB plan	Cannulation (RtFA: 18Fr, Asc Ao: 14Fr, RtFV: 23/25Fr)
	Targeting CI: 2.0–2.6 L/min/m^2^, mABP: 60–70 mmHg
	Targetting rectal temperature: 26 °C
	Systemic hyperkalemia combined with circulatory arrest
	Repeatedly circulation arrest: for up to 5 min
	Prevent oliguria management: 10 mL/h of continuous furosemide
	Targetting blood potassium level: 8.0–10.0 mEq/L
	Dialysis: Gravity drainage hemofiltration method
Operative CPB management	CI: 2.4 L/min/m^2^
	mABP: 60 mmHg
	Nadir rectal temperature: 26 °C
	Circulation arrest time: 25 min
	Cardiac arrest time: 90 min
	CPB time: 394 min
Postoperative outcomes	ICU stay: 3 day
	Ventilation time: 37 h
	Hospital stay: 16 days
	Major complications: None

Following induction of general anesthesia, the patient underwent redo-MVP via right thoracotomy procedure. CPB was established with a venous cannula 23/25Fr (MICS Cannulae; LivaNova, Tokyo, Japan) placed via the right femoral vein (FV) and an arterial cannula 18Fr (PCKC-A, MERA, Tokyo, Japan) placed in the right FA. Because it is possible to ensure total flow, we chose 18Fr in FA in response to what may arise when Asc cannulation causes any troubles. Until Asc Ao cannulation, CI (cardiac index) was kept at less than 1.0 L/min/m^2^ to prevent cerebral plaque embolism. After an arterial cannula 14Fr (PCKC-A, MERA, Tokyo, Japan) was placed in the Asc Ao, CPB was managed from 2.0 to 2.6 L/min per m^2^ of the target CI. Asc Ao cannulation was performed two-window technique. Phenylephrine and noradrenaline were administered to maintain mean arterial blood pressure (mABP) above 60 mmHg. A CDI Blood Parameter Monitoring System 500 (Terumo, Tokyo, Japan) was recalibrated every 30 min, and an arterial blood gas sample was also checked every 30 min. The patient was cooled to 26 °C. To obtain cardiac arrest, 800 mL of the hyperkalemia solution (500 mL of bicarbonate ringer solution with 50 mL of KCL 10 mEq/L, 20 mL of MgSO_4_, and 100 mg of 2% lidocaine) administered bolus infusion from CPB circuit. To maintain cardiac arrest, we continuously infused hyperkalemia solution, and the target of a blood potassium level was managed at 9 mEq/L. Due to the influence of aortic regurgitation, it was difficult to get a good vision. Therefore, we performed MVP combined with circulation arrest. When a left ventricular vent through the MV was useful in preventing aortic valve release, we started rewarming the temperature and lowering potassium levels in the blood by using dilutional ultrafiltration (DUF) and administering continuous furosemide (45 mL of 20% mannitol + 50 mg of furosemide) at 10 mL/h. Weaning from CPB was performed using inotropes (5.5γ of dobutamine and 0.03γ of noradrenaline). Mean CI and mABP were 2.4 L/min/m^2^ and 60 mmHg. Circulation arrest time, cardiac arrest time, and CPB time were 25 min, 90 min, and 394 min, respectively. Due to difficulty stopping bleeding, it became a long perfusion time. The duration of mechanical ventilation and length of stay in the intensive care unit (ICU) was 37 h and 3 days, respectively. The postoperative course was uneventful without cerebral infarction and he was discharged 16 days. Informed consent to report patient information and images was obtained.

## Discussion

In recent years, a meta-analysis reported that minimally MICS for redo cases has many advantages over median sternotomy [1]. In our institution, we actively employ totally endoscopic MICS for redo cases, aiming to mitigate risks and optimize patient outcomes [9]. However, re-operative valve surgery is acknowledged to be more complex and has increased morbidity and mortality rates [10]. Moreover, patients with atherosclerotic disease such as intravascular thrombus or severe calcification are more likely to have postoperative cerebral infarction by using retrograde perfusion via the FA [5, 11]. MICS through right minithoracotomy is often established CPB by commonly used femoral cannulation [2, 3]. In patients with atherosclerotic and artheromic aorta, antegrade perfusion using axillary cannulation, or Asc Ao is performed to prevent post-operative cerebral complications [5, 7, 12].

Few studies have examined the clinical outcomes comparing combined with central cannulation and FA cannulation in MICS. Huang et al. reported that 96.9% (317/327) of the patients undergoing femoral and axillary artery cannulation survived. In addition, their study indicated that incidences of permanent neurologic dysfunction, renal insufficiency, liver failure, and lower limb ischemia seldom occurred [4]. There are no perfusion strategies of MICS guidelines for atherosclerotic and artheromic aorta. In our institution, combined with ascending aorta or axillary artery and FA perfusion have selected if any of the following preoperative enhanced CT scan criteria were satisfied anywhere in the aorta or iliac arteries: thrombosis thickness >4.0 mm, calcification present in the total circumference. In our institution, when inserting aorta cannulation, we employ the two-widow technique. A 14Fr central cannula pass through the cranial window, in two-window technique. Bleeding is controlled by intermittent snaring of the tourniquet [13]. This technique is safer than single central cannulation because we keep the femoral cannula as a backup during cannulation and de-cannulation. However, there is no evaluation of the mixing zone when antegrade and retrograde perfusion are combined during CPB. To understand the mixing zone in the case of the Asc Ao and FA perfusion, we conducted a simulation study using an arteriovenous circulation system [8]. This 3D model successfully reproduced near human hemodynamics. This 3D model simulator was developed as a percutaneous coronary intervention. In this simulation study, we have evaluated the mixing zone by inserting Asc and FA cannulation. Priming solutions for CPB used 25% glycerol. Contrast agent (Hexabrix, serial No 15HJ031, Guerbet LLC) infused with 10 mL of undiluted solution from the femoral cannula side. And, the study was performed without the aortic cross-clamping, like this case, reproduced under cardiac arrest (Video 1). The simulation result illustrated the mixing zone when changing the cannulation size combination’s flow from 3.0 L/min to 5.0 L/min (Figures 2–4). In this case, by using 14Fr in the Asc Ao and 18Fr in the FA, the mixing zone of 3.0 L/min (FA side flow: 1.0 L/min, Asc Ao side flow: 2.0 L/min) was recognized as being in the descending aorta, but the mixing zone of 4.0 L/min (FA side flow: 1.4 L/min, Asc Ao side flow: 2.6 L/min) and 5.0 L/min (FA side flow: 1.6 L/min, Asc Ao side flow: 3.4 L/min) approached near the aortic arch (Figures 2a–2c). In this case, however, we did not measure the flow rate ascending the Asc Ao and FA, and from the result of the simulator, it is thought that the mixing zone was placed in the descending aorta as mean CI 2.0 L/min/m^2^ (3.18 L/min). For the combination of 14Fr in the Asc Ao and 16Fr in the FA, the mixing zone was placed at the descending aorta, and even at each flow rate, there were no major changes in the mixing zone. At 3.0 L/min, FA side flow: 1.3 L/min, Asc Ao side: 1.7 L/min. At 4.0 L/min, FA side flow: 1.7 L/min, Asc Ao side: 2.3 L/min. At 5.0 L/min, FA side flow: 2.1 L/min, Asc Ao side: 2.9 L/min (Figures 3a–3c). When 14Fr in the Asc Ao and 20Fr in the FA were combined, the mixing zone was recognized at the aortic arch, and it remained the same place even when the flow rate was changed. At 3.0 L/min, FA side flow: 0.8 L/min, Asc Ao side: 2.2 L/min, At 4.0 L/min, FA side flow: 1.0 L/min, Asc Ao side: 3.0 L/min, At 5.0 L/min, FA side flow: 1.3 L/min, Asc Ao side: 3.7 L/min (Figures 4a–4c). From these results, when changing the flow rate, the change was larger in 16Fr and 18Fr than in 14Fr. However, the smaller the size of the Asc Ao was than the size of the FA, the mixing zone tended to be closer to the aortic arch. Therefore, it was considered that our strategy for cannulation’s size choice for atherosclerotic and artheromic aorta is chosen until one size was up of FA cannulation size when performing perfusion Asc Ao and FA. Moreover, to prevent cerebral infarction in an atherosclerotic and artheromic aorta, the flow measuring of the Asc Ao and FA might be necessary. In the combination of 14Fr and 20Fr, the mixing zone substantially approached the aortic arch. However, this simulation is limited because of not considering the ventral branches such as the celiac artery, renal artery, and superior mesenteric artery. Therefore, the mixing zone might be possible to become lower than this simulation study. Also, we investigated and could not find the mixing zone in combination with antegrade and retrograde perfusion, and it needs to be investigated further.

## Data Availability

All available data are incorporated into the article.

## Supplementary material

The supplementary material of this article are available at https://ject.edpsciences.org/10.1051/ject/2024036/olm. *Video 1.* The difference in the arterial cannula size and variation of the mixing zone.
